# Supplementary material for: High-fidelity microsecond-scale cellular imaging using two-axis compressed streak imaging fluorescence microscopy
Source: ArXiv. 2025 Oct 23:arXiv:2412.16427v2. Preprint. [Version 2] (PMC12633632)
Supplement: Supplement 1 [file NIHPP2412.16427v2-supplement-1.pdf]

Appendix A Streaked illumination

The fundamental concept used in two-axis compressed streak imaging (TACSI) to reduce motion blur was derived from a streaked illumination method pioneered in 1893 by Boys et al. developed for high speed bullet imaging[34]. Figure A1 shows an artistic rendering of the key components from the landmark 1893 experimental setup. The wires were not drawn to simplify the diagram. In the landmark experiment, a flash was generated when the bullet completed a circuit between the Leyden jar and a spark gap electrode. By translating the illumination source, the exposure duration can be shortened substantially. In TACSI, the bullet is analogous to the translating

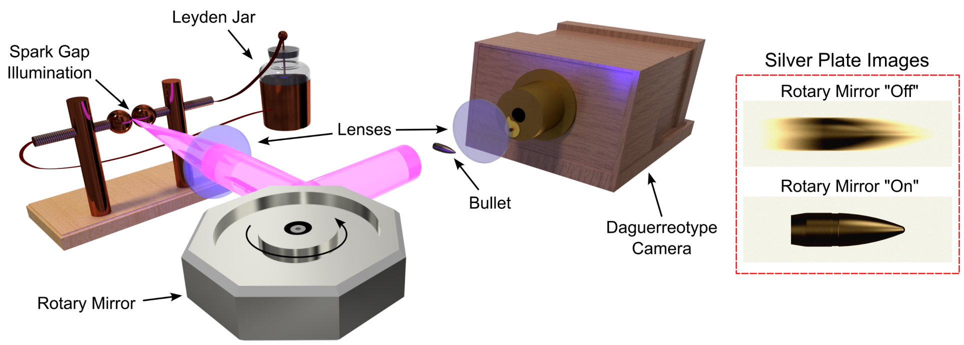

Fig. A1 Artistic rendering of the 1893 bullet imaging experimental setup. The Leyden jar provided the voltage necessary to generate a flash at the spark gap electrodes. The rotating mirror streaked the flash across the bullet, resulting in a substantially shorter exposure duration. Simulated daguerreotype silver plate images show the differences between images with the rotary mirror on or off.

image of the coded aperture (CA). By translating a continuously illuminated object, the elements of the mask are exposed for a shorter duration than would be possible without translation.

Appendix B Compressed streak imaging microbead simulations

Single frames from simulated single- and two-axis reconstructed videos are compared to their ground truth in Figure B2. Table B1 shows improvements in peak signal to noise ratio (PSNR), structural similarity index measure (SSIM), and the bit-utilization ratio (BUR) derived from reconstructions from simulated microbeads using the plug-and-play alternating direction method of multipliers (ADMM-PnP). This improvement was obtained with the object speed set 10-fold higher than the streak speed. Additionally, the resulting compression ratio was reduced by a factor of 10.

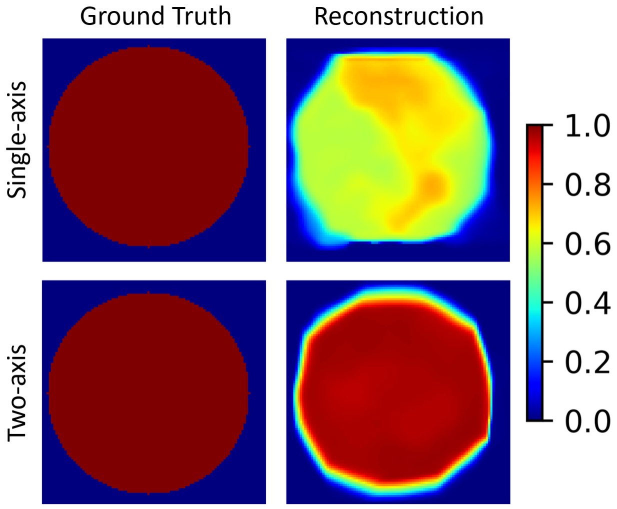

Fig. B2 Comparison between normalized frames from reconstructed single- and two-axis videos and their ground truth.

Table B1 Comparison between single- and two-axis microbead simulation metrics.

Metric	Single-axis	Two-axis
PSNR (dB)	15.9	18.3
SSIM	0.524	0.740
BUR (%)	3.62	15.8
CR	50.8	5.05

Appendix C Cave dataset compressed hyperspectral simulations

Improvements in reconstructed video quality may extend beyond CSI to compressed hyperspectral imaging (CHI). Simulated streak images were derived from 31 scenes within the Cave Hyperspectral Dataset[54]. An example of the single-axis and TACSI compressed streak images for the ”Jelly Beans” CAVE scene is shown in Figure C3, along with the CA and a ground truth frame. Comparisons between the original image, single-axis, and two-axis at 550 nm (yellow) are shown in Figure C4. An RGB image is included to assist with interpreting the color intensities.

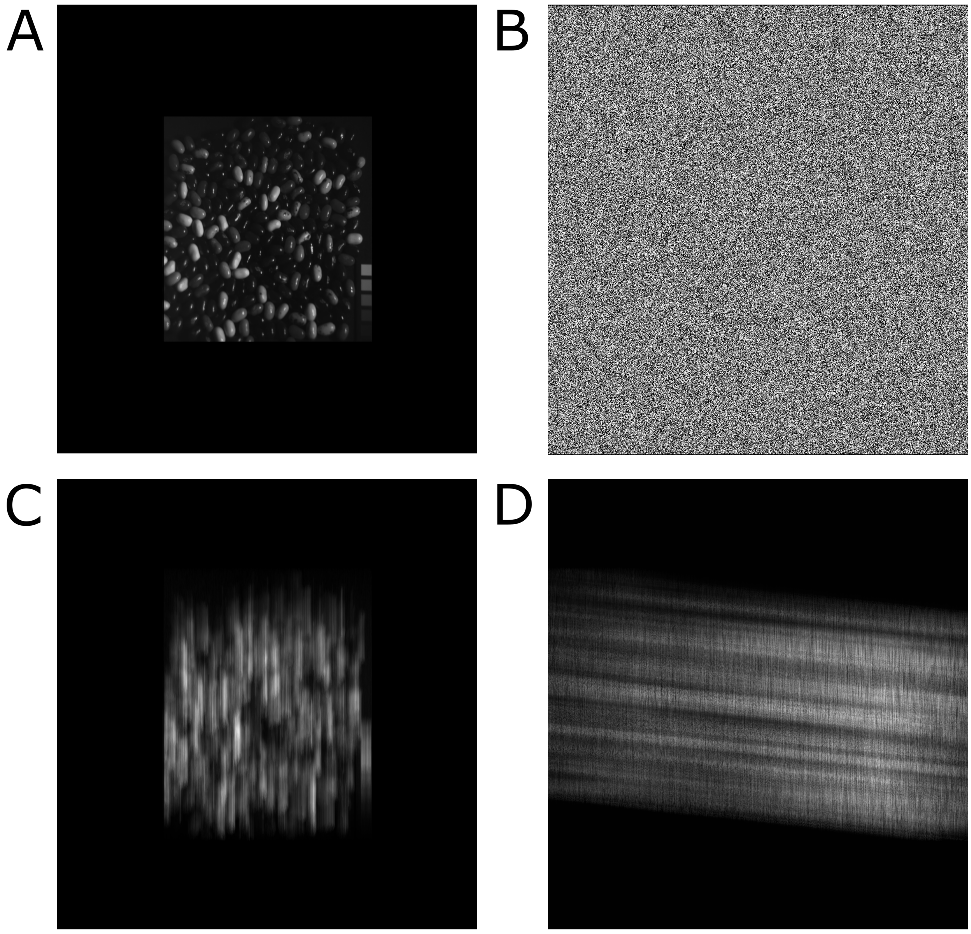

Fig. C3 Jellybeans streak simulation dataset. (A) A single frame from the ground truth video. (B) The coded aperture used in the single- and two-axis streak procedures. (C) The single-axis compressed streak image. (D) The two-axis compressed streak image.

Two-axis reconstructions require an extra translation step to nullify the induced object movement. This step results in a static object and simultaneously translates the positions of the reconstruction artifacts. Because the artifacts move from frame to frame, they can be removed using a temporal median filter.

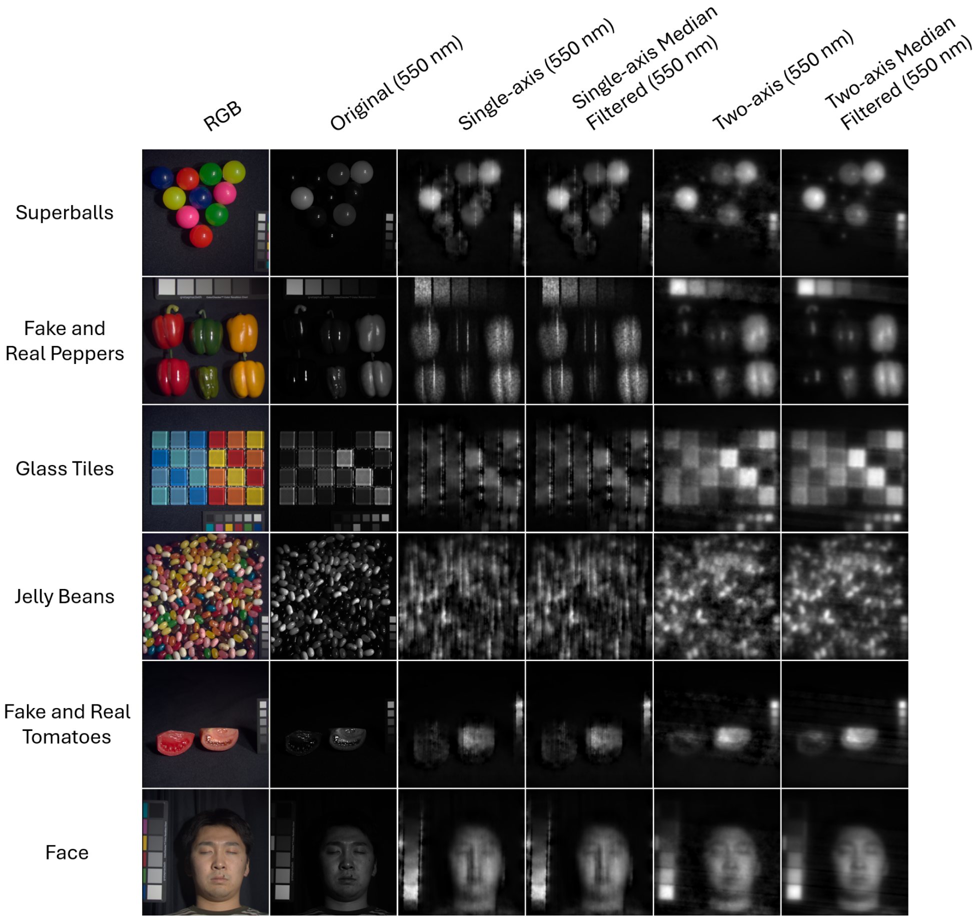

Fig. C4 Compressed hyperspectral simulations using complex scenes. Scenes from the Cave Dataset were used to generate single- and two-axis compressed hyperspectral simulations. The original image at 550 nm (yellow) is compared to the 550 nm frames from the reconstructed single- and two-axis videos. An RGB image is included to assist with color intensity comprehension. The temporal median filtered 550 nm image for each modality shows greater artifact reduction for two-axis images.

Face detection was performed using the RetinaFace[65–67] on the Face scene from the Cave Hyperspectral Dataset. Figure C5 shows the results from applying the face detection network to the ground truth image, single-axis reconstruction, and two-axis reconstruction. The network is unable to detect facial features in the single-axis reconstruction, whereas the two-axis reconstruction accurately predicts the location

of the face, eyes, nose, and corners of the mouth. Retina Face was able to detect facial features in the single-axis reconstructions after applying a custom filter to the image FFT. The vertical (or diagonal) lines were masked along with the high frequency features in both reconstructions.

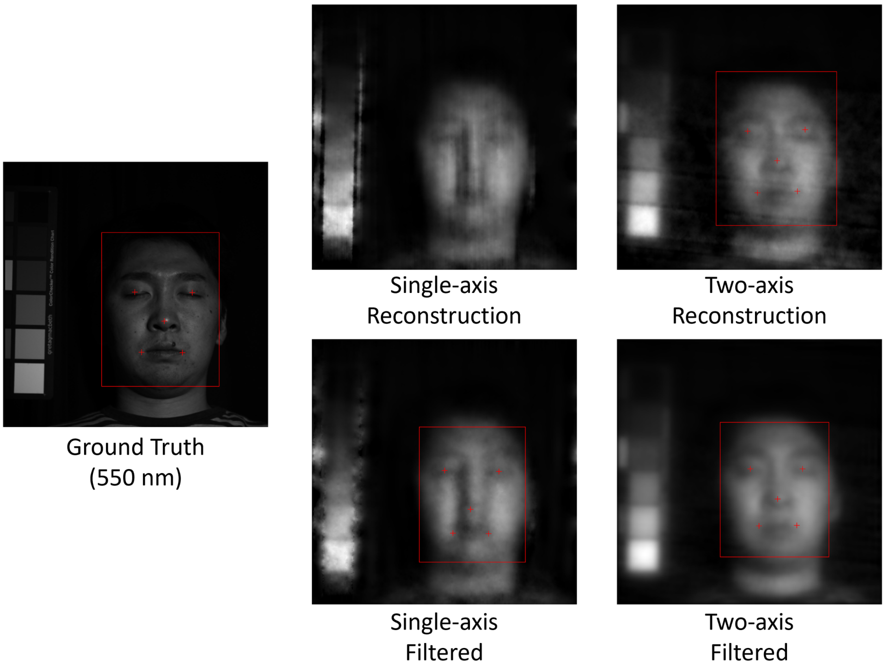

Fig. C5 Two-axis reconstruction enables face-tracking. The Retina Face face tracking algorithm was applied to the 550 nm ground truth, raw reconstructed single-axis, raw reconstructed two-axis, and temporal median filtered images for each modality with custom masks applied to block diagonal or vertical striations. Red bounding boxes surround the faces, with markers positioned at the eyes, nose, and corners of the mouth, in all images except for the raw single-axis reconstruction.

Appendix D Closed slit streak imaging

Prior to acquiring CSI images of CHO-K1 cells, electric pulse timing and strength was visualized with a closed slit streak image to ensure that the pulses occurred during the acquisition window and that the pulses would not cause the cells to move. Figure D6 shows a representative streak profile from a CHO-K1 cell containing Fluovolt voltage sensitive dye. Signal at the anode (blue) increases while signal at the cathode (red) decreases upon pulse delivery. The pulse strength was insufficient to cause the cell to move, as indicated by the straight cell edges.

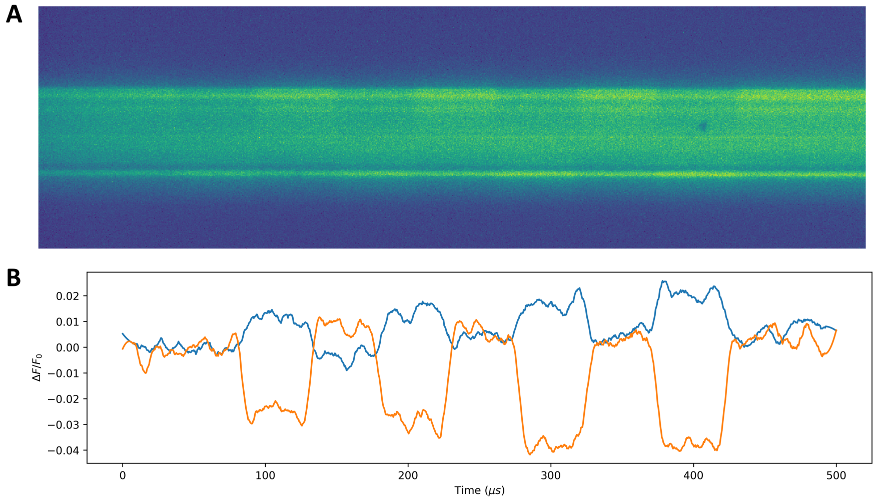

Fig. D6 Closed slit streak image. Image of a CHO-K1 cell loaded with Fluovolt voltage sensitive dye during electric pulse application with the mechnical slit closed and the coded aperture removed. The intensity at the membrane proximal to the anode increased in intensity, while the membrane proximal to the cathode decreased in intensity. The straight horizontal cell edges demonstrate that the cell did not move during the pulse.

Appendix E TACSI Compression Ratio Derivation

Controlling the portion of the camera sensor over which the digital spatiotemporal streak signal is acquired has an effect on the signal's compression ratio, defined generally as in Equation (E1)[24]:

$$CR = \frac{N_x N_y N_t}{N_{s'}}, \tag{E1}$$

where N_x, N_y, and N_t are the number of pixels required to convey the spatial information along the x and y coordinate axes and the time information of a full resolution video. $N_{s'}$ refers to the number of spatial pixels after the video has been compressed into a single streak image. Because the spatial information and the temporal

information mix, the total number of camera pixel rows P_y must be constrained by:

$$P_y \geq N_y + N_t - 1. \tag{E2}$$

This implies that the compression ratio definition treats the extent of the signal and not that of the camera sensor.

The following is a derivation of the mathematical description of the TACSI compression ratio. Since many scenes are characterized by objects contrasted against dark counts, a general description of the scene should have an amorphous geometry. Using a bounding box surrounding the region of interest may result in an overestimate of the CR. When discussing this topic, the streak velocity v_s will be used to refer to the translation rate of the coded aperture image formed at the camera sensor along the sensor's vertical axis. The term object velocity will be used to describe the speed and direction of motion of the image of the object under investigation at the camera sensor plane, with coordinates defined in relation to the horizontal and vertical sensor axes. Figure E7 depicts the path traced by a blob shaped object defined by the object's velocity and the streak velocity.

The first challenge in describing compression ratio is determining the constraints. The post-reconstruction frame rate can provides a useful constraint for determining the correct number of frames in the high resolution image. This is necessary because the full resolution image is often hypothetical under empirical conditions. The frame rate of a compressed streak imaging system is defined in Equation (E3):

$$FPS = \frac{v_s}{p}\,, \tag{E3}$$

where v_s is the streak velocity and p is the pixel pitch of the camera.

The surface area of the streak in square pixels can be determined from Equation (E4):

$$N_{s'} = \frac{w_{\perp} t \sqrt{v_o^2 + v_s^2} + s_o}{p^2}\,, \tag{E4}$$

where s_o is the surface area of the object in square meters, $w_{\perp}$ is the width of the object along an axis perpendicular to its trajectory, and t is the streak duration. The number of image frames in the high resolution video can be found using Equation (E5):

$$N_t = \frac{v_s t}{p}\,, \tag{E5}$$

and the number of spatial pixels as in Equation (E6):

$$N_s = \frac{s_o}{p^2}\,. \tag{E6}$$

We can now use Equation (E7) to determine the compression ratio of the streak image:

$$CR = \frac{N_s N_t}{N_{s'}} = \frac{s_o v_s t}{p(w_{\perp} t \sqrt{v_o^2 + v_s^2} + s_o)}\,. \tag{E7}$$

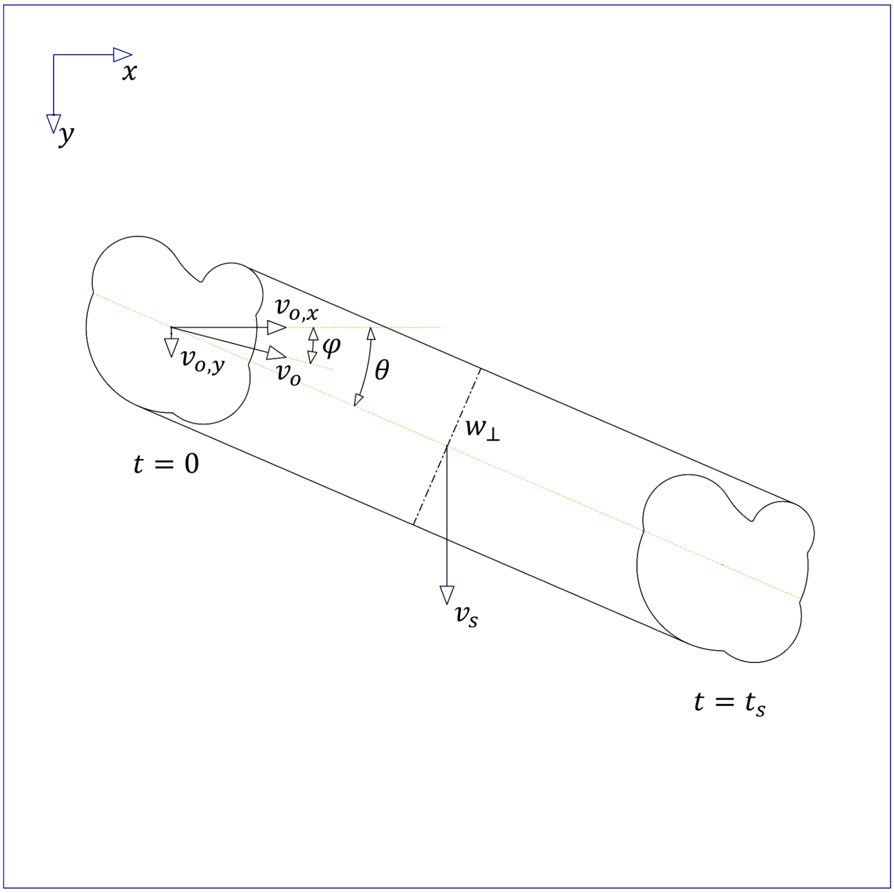

Fig. E7 Streak imaging conceptual model. The compression ratio can be developed by considering the surface area exposed by a blob with a object velocity v_o from time $t = 0$ to $t = t_s$. The streak velocity v_s is the rate at which the image of the CA is propagated with respect to the sensor's vertical axis. The vertical extent of the object along normal to its trajectory is given by $w_\perp$. While this model applies to any arbitrary object velocity angle φ, the TACSI system translates the image of the sample along the horizontal axis of the sensor ($\varphi = 0$).

Let's assume that we want to recover a 200 kFPS video. Then, with our camera's 2x2 binned pixel pitch of 22 μm, we would need a 4.4 m/s streak velocity along the y-axis based on Equation (E3). Further assume that the object is a circular bead with a 2 mm diameter at the camera plane, moving with an object velocity of 44 m/s (10x) along the x-axis. The streak interval is determined by the time required for the bead to travel twice its diameter along the direction of the total velocity, considering both the object and streak velocities. This duration represents the maximum local frame density that arises as the object is convolved with itself across successive time points. Using Figure E7 as a reference, the overlap time can be generally computed with

Equation (E8):

$$t = \frac{2d_\theta}{\sqrt{v_{o,x}^2 + (v_{o,y} + v_s)^2}}, \tag{E8}$$

where d_θ indicates the diameter of the bead taken along the axis parallel to the total velocity vector, with the magnitude of the total velocity indicated in the denominator of Equation (E8). In the case of our TACSI system, the object velocity travels perpendicularly to the streak velocity ($\phi = 0$), allowing for $v_{o,x} = v_o$ and $v_{o,y} = 0$. The expression then simplifies to Equation (E9):

$$t_{\phi=0} = \frac{2d_\theta}{\sqrt{v_o^2 + v_s^2}}. \tag{E9}$$

Using Equations (E7) and (E9) along with the bead geometry, pixel pitch, object velocity, and streak velocity specified earlier in this example, the compression ratio for the object moving horixontally at 44 m/s is approximately 5.1. If we then set the object velocity to 0 m/s, the compression ratio increases to 51.3. This indicates a remarkable 10-fold improvement in the compression ratio in the case of the moving object compared to the stationary object.

Figure E8 shows the compression ratio as a function of the ratio between the object speed and the streak speed. These results assume that the camera sensor is always wide enough to encode the entire streak interval. A streak ratio of 0 implies the object is stationary, while a ratio 10 would correspond to a 44 m/s horizontal object velocity.

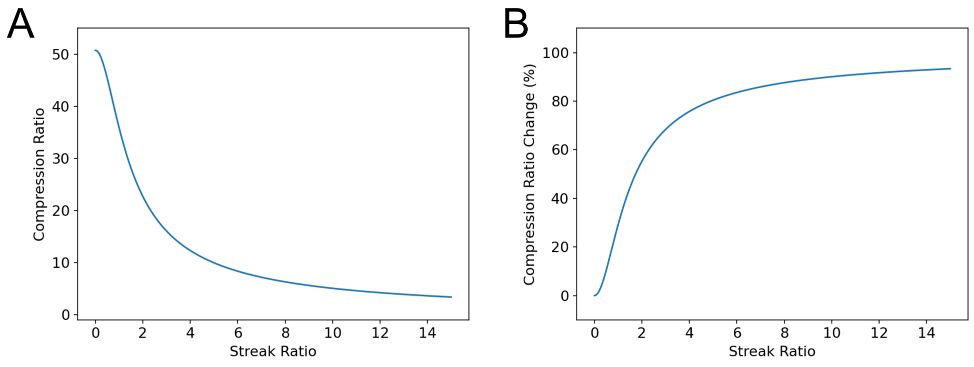

Fig. E8 Relationship between the compression ratio and the streak ratio. Compression ratio trends can be observed for a circular object with a 2 mm diameter at the camera sensor plane. (A) shows a decreasing compression ratio as the ratio between the object speed and the streak speed increases. (B) shows the compression improvement as a percent with respect to the maximum.

Appendix F Discretized TACSI Compression Ratio

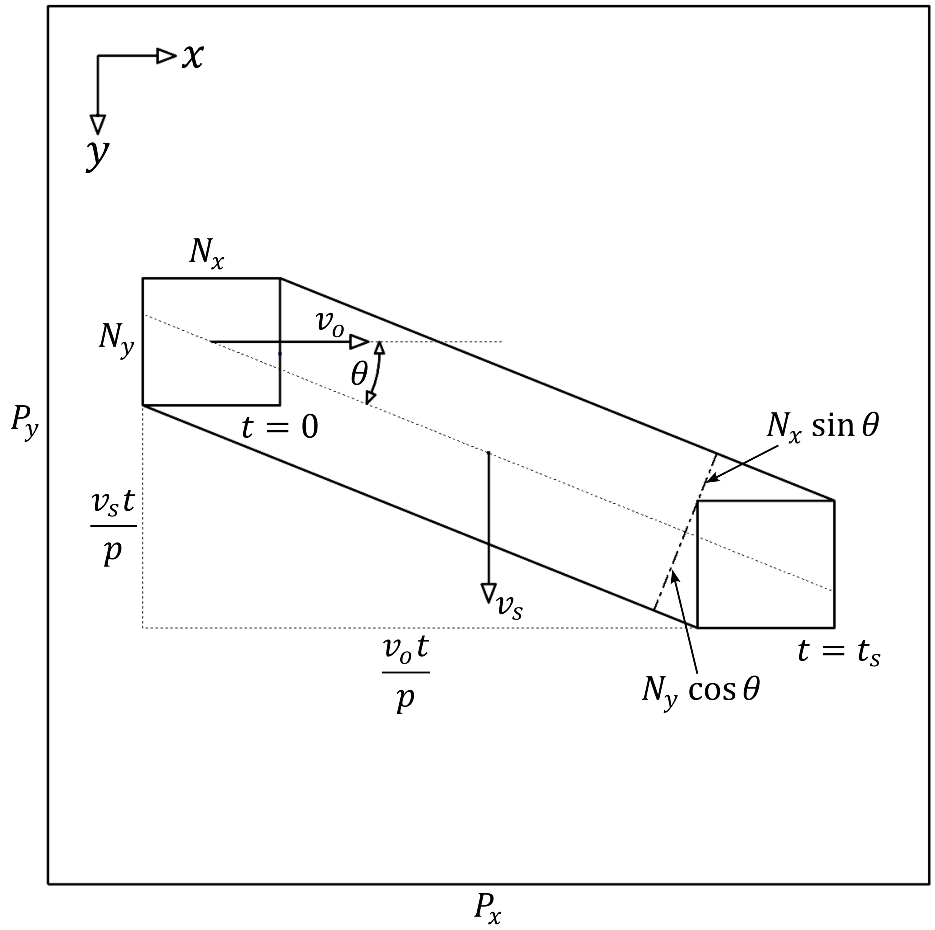

Fig. F9 Streak imaging conceptual model for a rectangular scene. A discrete compression ratio model for a scene with a rectangular scene geometry can be developed by considering the model depicted above. The box that defines the perimeter of the model represents the imaging sensor.

The compression ratio of a single-axis compressed streak image can be calculated using Equation (F10)[24]:

$$CR = \frac{N_x N_y N_t}{N_x(N_y + N_t)}. \tag{F10}$$

The TACSI compression ratio, given by Equation (E7), can be directly compared to Equation (F10) by discretizing the variables and adopting a rectangular geometry.

Starting from the general form of the TACSI streak surface area in Equation (E4), it follows that:

$$\begin{aligned} N_{s'} &= \frac{w_\perp t\sqrt{v_o^2 + v_s^2} + s_o}{p^2} \\ &= \frac{w_\perp}{p} t\sqrt{\left(\frac{v_o}{p}\right)^2 + \left(\frac{v_s}{p}\right)^2} + \frac{s_o}{p^2} \\ &= \frac{w_\perp}{p}\frac{v_s t}{p}\sqrt{r^2+1} + \frac{s_o}{p^2} \\ &= \frac{w_\perp}{p} N_t\sqrt{r^2+1} + \frac{s_o}{p^2}. \end{aligned} \tag{F11}$$

The perpendicular width of the streak for a scene with a rectangular geometry is:

$$\begin{aligned} \frac{w_\perp}{p} &= N_x sin\theta + N_y cos\theta \\ &= N_x sin\left(tan^{-1}\left(\frac{1}{r}\right)\right) + N_y cos\left(tan^{-1}\left(\frac{1}{r}\right)\right). \end{aligned} \tag{F12}$$

For convenience, r is defined as the ratio between the object and streak speed:

$$r = \frac{v_o}{v_s}, \tag{F13}$$

and the surface area of the rectangular scene is:

$$\frac{s_o}{p^2} = N_x N_y. \tag{F14}$$

The discrete form of the TACSI compression ratio can now be written as:

$$CR = \frac{N_x N_y N_t}{N_t\left[N_x sin\left(tan^{-1}\left(\frac{1}{r}\right)\right) + N_y cos\left(tan^{-1}\left(\frac{1}{r}\right)\right)\right]\sqrt{r^2+1} + N_x N_y}. \tag{F15}$$

While the number of recovered image frames in a single-axis streak image will be limited by the height of the scene and the number of row pixels:

$$N_t \leq P_y - N_y, \tag{F16}$$

TACSI requires considering constraints along the horizontal and vertical axis of the imaging sensor:

$$N_t \leq min\left\{P_y - N_y, \frac{P_x - N_x}{r}\right\}. \tag{F17}$$

Appendix G Alternative Streak Methods

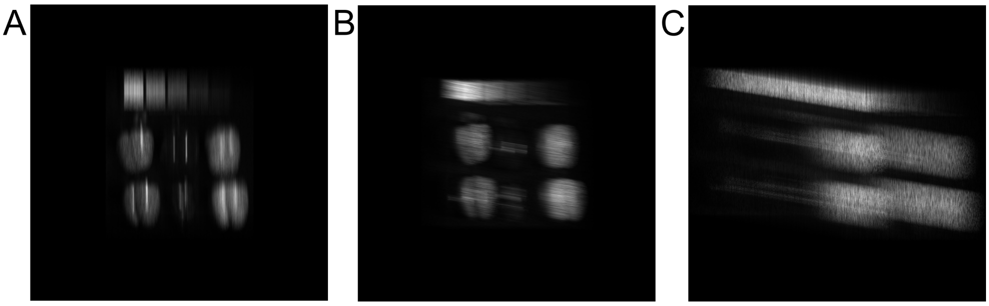

Fig. G10 Comparison of simulated compressed streak images generated with (A) conventional single-axis, (B) a two-axis galvo-scanner positioned between the CA and the camera, and (C) TACSI. The streak speed was held constant for all methods.

Figure G10 shows simulated compressed streak images acquired using a hypothetical method in which a two-axis galvo-scanner is positioned between the coded aperture and the camera, conventional single-axis streak, and TACSI. The streak speed was held constant for all methods. The relationship between frame rate and streak speed is described by SI Appendix, Equation (E3). Both single-axis and TACSI ensure that temporal encoding is along the y-axis in Figure G10A and G10C. The two-axis galvo-scanner results in temporal encoding along the diagonal as seen in Figure G10B. Since a two-axis galvo-scanner would shear the mask along a diagonal, the concept of pixel pitch would need to be redefined. Determining this definition is outside of the scope of this manuscript. Furthermore, single-axis streak and the compressed streak image obtained with the two-axis galvo have identical motion blur because the object is not moving. The inability to decrease motion blur and compression ratio can be observed in Figure G10B, where the coded aperture elements are not distinguishable. For TACSI, because the streak speed does not depend on the speed of the object, it is possible to adjust the object speed while maintaining the frame rate. This allows the compression ratio and motion blur to be reduced simultaneously. For the compressed streak image in Figure G10C, the object speed was 6-fold faster than the streak speed. It is important to note that the coded aperture elements in Figure G10C can be distinguished. Similarly, no advantage can be gained by simply rotating the camera or galvo-scanner, and rotating the camera with respect to the CA would cause a mismatch between the CA elements and the camera pixels.
